# Design and Fabrication of a Wideband Cymbal Transducer for Underwater Sensor Networks

**DOI:** 10.3390/s19214659

**Published:** 2019-10-27

**Authors:** Hayeong Shim, Yongrae Roh

**Affiliations:** School of Mechanical Engineering, Kyungpook National University, Daegu 41566, Korea; hiyo3@naver.com

**Keywords:** cymbal transducer, optimization, finite element analysis

## Abstract

Cymbal transducers are characterized by a high mechanical quality factor and low power efficiency. The research conducted so far on cymbal transducers has focused on improving the power efficiency and structural stability, but modern underwater sensor network systems need transducers to have a wide frequency bandwidth as well. In this study, a wideband cymbal transducer was designed to fill that need. First, the effect of various structural parameters on the performance of the cymbal transducer was analyzed with emphasis on the bandwidth using the finite element method. Based on the analysis results, the structure of the cymbal transducer was optimized to have the widest possible bandwidth while maintaining its transmitting voltage response (TVR) level over a typical power requirement as well. The validity of the design was verified by fabricating a prototype of the optimized cymbal transducer and comparing its measured performance with the design.

## 1. Introduction

Underwater sensor network (UWSN) technology enables underwater exploration by combining sensor technology with wireless technology, smart sensing, intelligent computing, and communication functions. UWSN uses 1D communication between sensor nodes fixed in water, 2D communication between gateway nodes and fixed nodes in water, 3D communication between nodes having different fixed-position depths, and 4D communication with movable sensor nodes [1]. This technology can be applied to various fields such as gas extraction, military surveillance, natural disaster forecasting, marine life habitat monitoring, and marine aquaculture [2]. The sensor nodes in these applications are acoustic transducers; high-performance acoustic transducers are essential to the proper operation of the UWSN. In particular, the acoustic transducers must have a wide bandwidth for high-speed data transmission [3]. The broadband characteristics of acoustic transducers not only enable mass data transmission in underwater communication systems but also improve detection accuracy in sonar systems.

A cymbal transducer is a miniature transducer developed from the class V flextensional transducer. It has a simple structure consisting only of a piezoceramic disk sandwiched between metal caps [4]. This transducer is a quite promising candidate for future UWSN systems for the following reasons. The cymbal transducer shares its principle of operation with the class V flextensional transducer; the displacement generated by the piezoceramic disc is amplified by the relatively flexible cap attached to the piezoceramic in an effect analogous to a lever [5]. The metal caps can be manufactured using a punching or machining process. Therefore, it is easy to fabricate and has all the inherent advantages of class V flexible transducers such as high power, low weight, and small volume [4,6]. In addition, the cymbal transducer can be used in various array forms due to its small size, which is suitable for large area and restricted volume transmit and receive arrays [7]. However, cymbal transducers generally exhibit high mechanical Q frequency characteristics and low power efficiency [8].

Since the invention of the cymbal transducer, many types of research have been conducted to advance its performance: the development of a concave cymbal transducer by Zhang et al. [9], a metal-ring-coupled cymbal transducer by Lin [10], and a bolt-reinforced cymbal transducer by Lucas et al. [11]. Narayanan and Schwartz developed a wagon wheel-type cymbal transducer [12]. Different materials have also been tried to improve the performance of the transducer [13,14]. Jenne conducted a study on the change of the resonant frequency in relation to the structural variables of the cymbal transducer, and observed the transmitting voltage response (TVR) and free field voltage sensitivity spectra of several structural variations of the cymbal transducer [15]. However, most of this research has focused on the enhancement of structural stability and the power efficiency of the transducer, not directly related to improving the bandwidth of the transducer that is required by modern UWSN systems.

Some work has been done to broaden the bandwidth when multiple cymbal transducers are used as an array [16,17,18,19,20]. The frequency characteristics of an acoustic transducer array are based on the properties of individual transducers constituting the array. However, the efforts to broaden the bandwidth of individual cymbal transducer have been very rare. Tressler and Newnham developed a double resonance cymbal transducer composed of asymmetric cap materials, and suggested the possibility of combining the two resonances to increase the bandwidth [21]. The impedance of an acoustical transducer at a frequency before a resonance is normally conductive while that at a frequency after the resonance is inductive [22]. Hence, at the frequency range between two resonances, the impedances from each resonance have opposite signs and thus are likely to cancel each other. This destructive coupling of different resonances makes a sharp notch at the range between the two resonance frequencies in the impedance spectrum as evidenced in [21]. Actually, this double resonance phenomenon occurs quite often due to irregularities in fabrication of the transducer, which is considered to be a significant drawback of the cymbal transducer. Hence, the double resonant structure does not contribute to increasing the bandwidth. Therefore, no notable work has been conducted so far on the design of wideband cymbal transducers.

In this study, we developed a wideband cymbal transducer that could maintain its TVR level over a typical power requirement as well. For this purpose, first, we analyzed the effect of various structural parameters on the transducer’s performance with emphasis on the bandwidth using the finite element method (FEM). Then, based on the analysis results, the structure of the transducer was optimized to have the widest possible bandwidth while satisfying given constraints on the TVR level and center frequency. The validity of the design was verified by fabricating a prototype of the optimized transducer and comparing its measured performance with the design.

## 2. Design of a Wideband Cymbal Transducer

**Figure 1:**
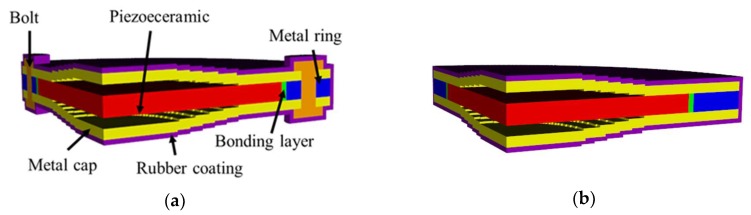
Typical cymbal transducer: (**a**) 3D model of a cymbal transducer with bolts, (**b**) without bolts [11].

A finite element analysis (FEA) model of a representative cymbal transducer was constructed to analyze the transducer’s acoustic characteristics using the commercial software PZFlex ®. Figure 1a illustrates the model. The transducer is comprised of a piezoceramic disc and a metal ring bonded to two metal caps of a cymbal shape [11]. The piezoceramic disc is glued to the metal ring as well, and the metal caps are bolted again to the metal ring. The piezoceramic is PZT-5A. The metal cap and ring are made of brass. In principle, the FEA model of the cymbal transducer should be three dimensional in order to incorporate the bolts. However, analysis with three-dimensional models is quite time-consuming. Therefore, we analyzed the acoustic characteristics of the transducer using 2D models ignoring the bolts.

**Figure 2:**
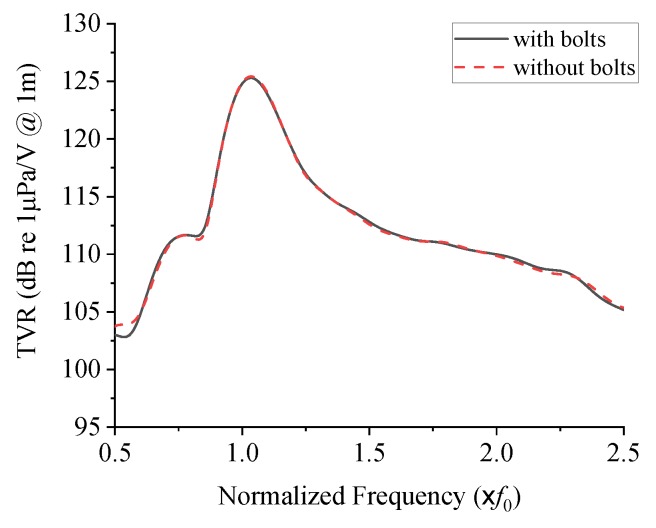
Transmitting voltage response (TVR) spectra of the cymbal transducer models with and without bolts.

To justify the ignoring, we checked the effects of the bolts on the acoustical characteristics of the cymbal transducer. Figure 1b shows a new 3D cymbal transducer model that has all the dimensions and geometry identical to those of Figure 1a but does not have the bolts. The underwater TVR spectra of the two transducer models were analyzed by the FEM and the results are compared in Figure 2, which shows very close agreement with each other. The decibel (dB) scale in the vertical axis was defined with respect to the reference sound pressure in water of 1 μPa [23]. 

**Figure 3:**
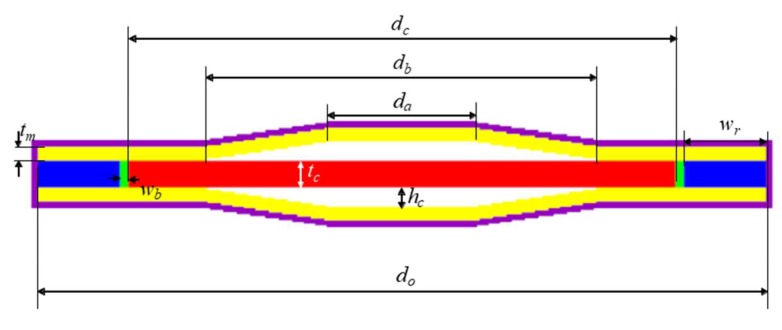
2D axisymmetric finite element analysis (FEA) model of the cymbal transducer.

**Figure 4:**
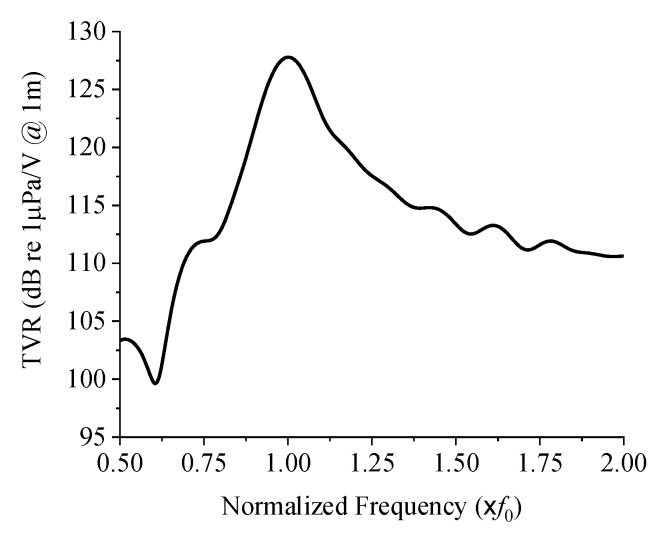
Underwater TVR spectrum of the initial cymbal transducer model.

As described in [11], the bolts were used only to improve the structural stability, not to influence the acoustical properties of the transducer. Hence, we could safely ignore the bolts for the analysis, and used the axisymmetric 2D model illustrated in Figure 3 in all the FEA afterwards. Figure 3 displays all the structural parameters of the transducer as well. The mesh size used in the simulation model is 0.07 mm. The adhesive layer between the cap and the combination of the piezoelectric disk and the metal ring was so thin that the adhesive layer was ignored in the simulation model. Table 1 shows the dimensions of the initial transducer model. The initial dimensions were the authors’ approximate estimate based on the results in precedent works [6,7,8,9] to achieve the center frequency and TVR level targeted in this work. For analysis of its underwater performance, the transducer was coated with a 0.25 mm thick rubber layer considering the practical situation and was immersed in water. The water’s periphery was enforced with a sound absorbing boundary condition. Figure 4 is the underwater TVR spectrum of the initial transducer model. The center frequency of the initial transducer is *f*_0_, the peak TVR level is 127.8 dB, and the −3 dB bandwidth is 0.14 *f*_0_ where *f*_0_ is 11.8 kHz, to which all the acoustical characteristics are to be normalized.

The purpose of this study was to design a cymbal transducer that exhibits the widest possible bandwidth with the TVR level over a certain threshold at a specified center frequency. To achieve this, the effects of the structural parameters in Table 1 on the performance of the transducer were analyzed using FEM. Through preliminary analysis of the TVR spectrum variation in relation to the structural parameters, the most influential parameters were determined as the thickness of the metal cap, the height of the cavity, and the apex and base diameters of the cavity. Dimensions of the piezoceramic disk, such as thickness and diameter, were fixed to constant values. The performance factors extracted from the TVR spectrum were center frequency, peak TVR level, and bandwidth. Based on the results of the analysis, the optimal combination of these parameters was to be determined to maximize the bandwidth of the transducer while meeting given performance constraints.

**Figure 5:**
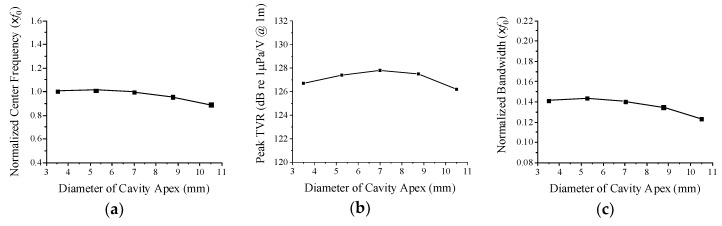
Variation of acoustic characteristics of the cymbal transducer in relation to the cavity apex diameter: (**a**) center frequency, (**b**) peak TVR level, (**c**) bandwidth.

Figure 5 shows the change in the transducer’s acoustic characteristics as a function of the cavity apex diameter. The diameter was varied around the initial dimension in Table 1. The center frequency and bandwidth tend to decrease when the apex diameter increases but the peak TVR level shows a maximum at a certain cavity apex diameter.

**Figure 6:**
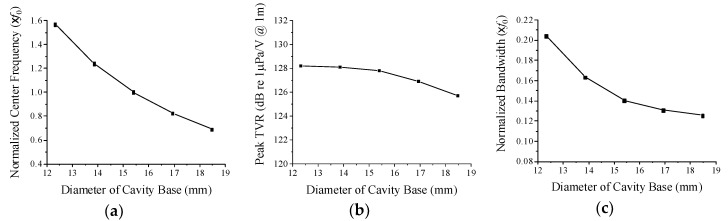
Variation of acoustic characteristics of the cymbal transducer with relation to the cavity base diameter: (**a**) center frequency, (**b**) peak TVR level, (**c**) bandwidth.

Figure 6 shows the variations in center frequency, peak TVR level, and bandwidth in relation to the cavity base diameter. When the cavity base diameter increases in the region around the initial dimension, the center frequency, peak TVR level, and bandwidth all tend to decrease. The slope of decrease is very steep for the center frequency and bandwidth but is mild for the peak TVR level. As the cavity base diameter increases, the effective area of the cap to bend increases accordingly, which leads to larger vibration of the cap. As a result, the overall stiffness of the transducer tends to decrease, lowering the center frequency, and the transient vibration of the cap is likely to increase reducing the bandwidth [24]. Hence, for a wider bandwidth, the cavity base diameter needs to be smaller on the condition that the center frequency reaches a desired value.

**Figure 7:**
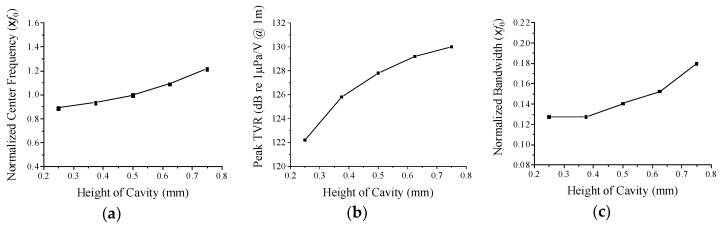
Variation of acoustic characteristics in relation to the cavity height: (**a**) center frequency, (**b**) peak TVR level, (**c**) bandwidth.

Figure 7 shows the change of the performance in relation to the cavity height; the center frequency, peak TVR level, and bandwidth all tend to increase in proportion to the cavity height. As the cavity height increases, the slope of the side surface of the cap becomes steeper, which increases the effective stiffness of the cap. Thus, the stiffer transducer has a higher center frequency. The higher stiffness is also likely to shorten the transient vibration of the cap, which results in a wider bandwidth. For a wider bandwidth, therefore, the cavity height needs to be larger as long as the center frequency can be controlled to a specific value. The increased space between the piezoceramic disc and cap seems to allow more vibration of the cap, yielding the higher peak TVR level.

**Figure 8:**
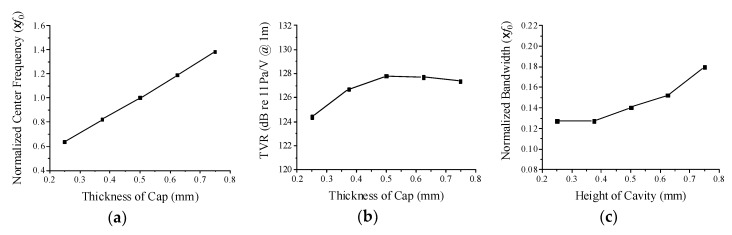
Variation of acoustic characteristics of the cymbal transducer in relation to the metal cap thickness: (**a**) center frequency, (**b**) peak TVR level, (**c**) bandwidth.

Figure 8 illustrates the change of the performance with the variation of the metal cap thickness. When the metal cap thickness increases, both the center frequency and the bandwidth tend to increase monotonically, but the peak TVR level shows a maximum at a particular cap thickness. The stiffness of the cap tends to increase in proportion to the thickness, which results in the increase of the center frequency. The higher stiffness also tends to shorten the transient vibration of the cap leading to a wider bandwidth. The reason why the peak TVR level shows a maximum at a particular cap thickness can be explained by the relationship between the displacement of the cap vibration and the center frequency of the transducer. The output sound pressure is proportional to the velocity of the cap vibration, which is the product of the center frequency and the displacement of the cap vibration [23]. The cap displacement decreases as the cap thickness increases. Then, the peak TVR level increases until the cap thickness increases up to a specific value because the center frequency increases more rapidly than the displacement decreases. However, when the thickness exceeds the specific value, the decrease of the displacement prevails over the increase of the center frequency, thereby decreasing the peak TVR level.

According to the results in Figure 5, Figure 6, Figure 7 and Figure 8, apex and base diameters need to be smaller while cavity height and cap thickness need to be larger to achieve a wideband cymbal transducer. However, these variables affect not only the bandwidth but also the center frequency and peak TVR level. Further, the effects of the structural variables are not independent of each other but are interrelated with each other. The purpose of this study is to determine the optimal geometry of the cymbal transducer that can provide the widest possible bandwidth at a specific center frequency while the peak TVR level is high enough to meet a given requirement. The initial transducer did not satisfy our requirement on the specific center frequency. However, many new combinations of the structural parameters were possible to achieve the specific center frequency. Therefore, there should be a clever way to determine the particular combination among them that could provide the maximum bandwidth. Determination of the particular combination was carried out through the optimization process described in the next section.

## 3. Optimal Design of the Wideband Cymbal Transducer Structure

The optimal transducer geometry was determined through FEA and statistical multiple regression analysis of the FEA results. The design scheme can reflect not only the individual but also all the cross-coupled effects of the structural variables. In comparison with conventional analytic and finite element methods, the present method can determine the detailed geometry of the cymbal transducer with great efficiency.

Based on the analysis results in the previous section, the design variables for the optimization were selected as the cavity height, cavity apex diameter, and base diameter. Although the cap thickness was an influential variable, its value was fixed to 0.5 mm in this work due to our constraint in machining the metal cap. The optimal combination of the three design variables was sought to maximize the bandwidth of the transducer by the method described in [25]. The objective function and constraints were set as Equation (1). Through literature survey, the targeted peak TVR level was set to be at least 125 dB, which is a very typical peak TVR level reported in previous works on cymbal transducers [4,8,26].

(1) $$\begin{array}{cc} \mathrm{Objective}\;\mathrm{function}: & \mathrm{maximize}\;\mathrm{the}\;\mathrm{bandwidth}\\ \mathrm{Constraints}: & ①\;\mathrm{peak}\;\mathrm{TVR}\;\mathrm{level}\geq \;125\;\mathrm{dB}\;②\;1.34f_{0}\;\leq \;\mathrm{center}\;\mathrm{frequency}\leq 1.38f_{0} \end{array}$$

As the first step of the optimization, the functional forms of the bandwidth, the peak TVR level, and the center frequency were derived in terms of the three design variables through multiple regression analyses of the FEA results in Figure 5, Figure 6 and Figure 7 [27]. We developed a second order multiple regression model that could consider the cross-coupled effects of the design variables by the product terms of the variables. With these regression functions, we could analyze the transducer performance for any combination of the variables without conducting the time-consuming FEA every time.

**Figure 9:**
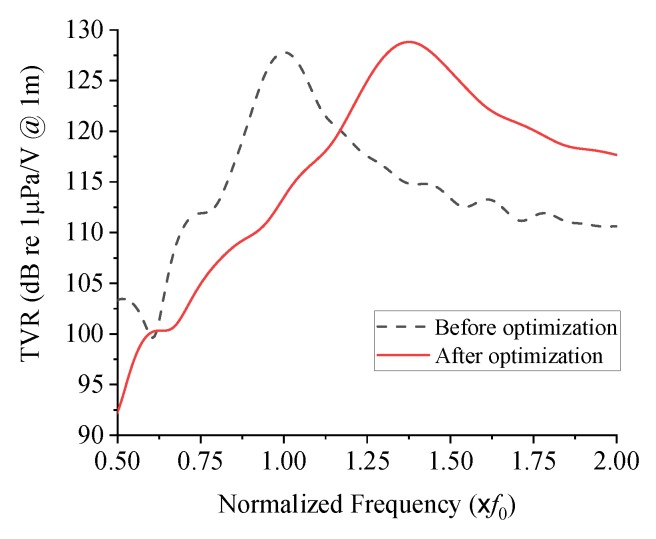
TVR spectra of the cymbal transducers before and after the structural optimization.

The derived functions were inserted into the objective function and constraints, and then the optimal combination of the design variables was searched by means of the OptQuest nonlinear programming (OQ-NLP) algorithm [28]. The results of the optimization are summarized in Table 2. Figure 9 illustrates the TVR spectrum of the optimized structure in comparison with that of the initial structure. Quantitative performances of the transducers before and after the structural optimization are compared in Table 3. The optimized transducer has a center frequency of 1.38 *f*_0_ and the peak TVR level of 128.8 dB, which safely satisfies the constraints. The −3 dB bandwidth has been increased from 0.14 *f*_0_ to 0.24 *f*_0_. In terms of fractional bandwidth (FBW), the FBW has been increased from 14.0% to 17.4%, i.e., increased by 24.3% from the initial value.

The cymbal transducer designed in this study has a wider fractional bandwidth than those in precedent works [6,7,8,16]. For instance, the most representative precedent cymbal transducer presented in [7] has the fractional bandwidth of about 13% working at 16.5 kHz with the peak TVR level of 124 dB. The present cymbal transducer can provide a wider bandwidth at a similar center frequency, thus a wider fractional bandwidth, with a higher TVR level than the precedent transducer.

## 4. Fabrication and Characterization of the Cymbal Transducer

**Figure 10:**
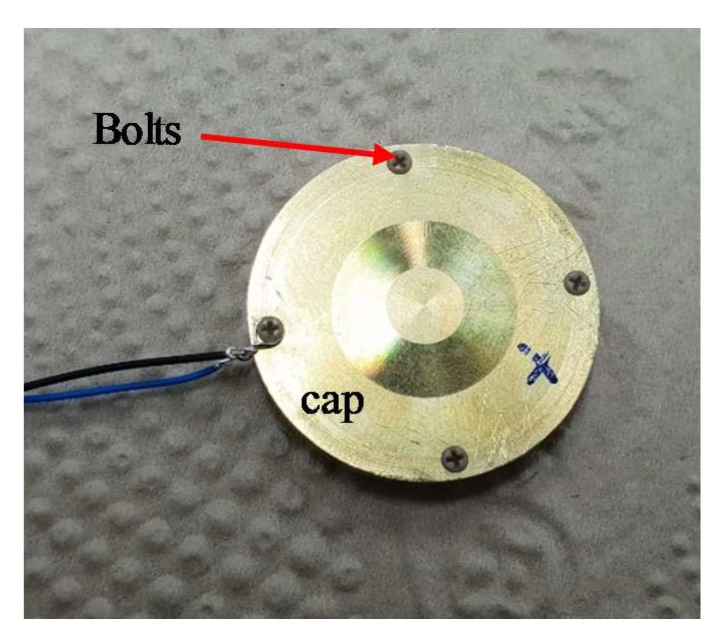
Photograph of the prototype cymbal transducer.

In order to verify the validity of the structure designed in Section 3, a prototype of the optimized cymbal transducer was fabricated and characterized. Following the FEA model, a prototype of the cymbal transducer was fabricated to have the dimensions in Table 1 and Table 2. The cavity height, cap base diameter, and cap apex diameter followed the optimized dimensions in Table 2 while all the other parameters followed the dimensions in Table 1. The piezoceramic was PZT-5A. The metal cap and ring were made of brass. The brass caps and ring were machined using a computer-numerical-controlled machine. The caps, piezoceramic disk, and ring were bonded using an adhesive (EB-106, EpoxySet, Inc., RI). Then, plastic bolts were used to further fix the brass caps to the ring as illustrated in Figure 1a. Figure 10 is a photograph of the prototype cymbal transducer before coating rubber. Electric wires were connected to the plastic bolt to connect the transducer to a power amplifier.

**Figure 11:**
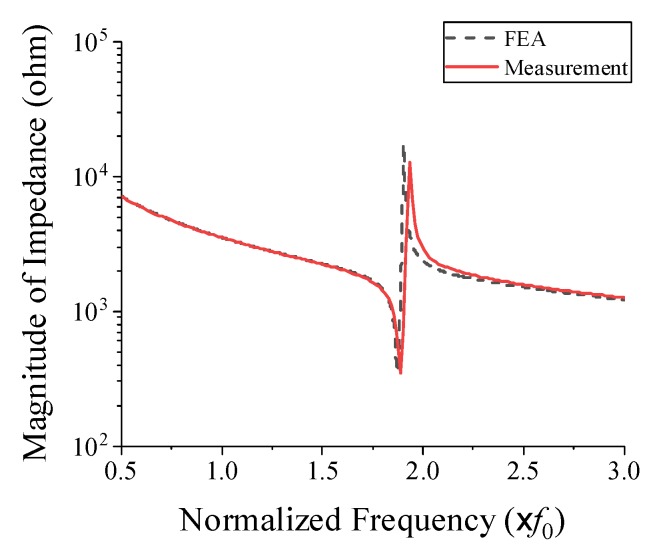
Comparison of the measured and simulated impedance in air of the prototype cymbal transducer.

The impedance of the prototype transducer in air was measured using an impedance analyzer (Agilent 4294A, Santa Clara, CA, USA). The measured impedance spectrum is shown in Figure 11 along with a simulated spectrum from the FEA. The resonant and anti-resonant frequencies from the measurement were 1.89 *f*_0_ and 1.93 *f*_0_, respectively, while those from the FEA were 1.87 *f*_0_ and 1.91 *f*_0_, respectively. This good agreement between the measured and simulated spectra verified that the prototype transducer conformed well to its structural design.

**Figure 12:**
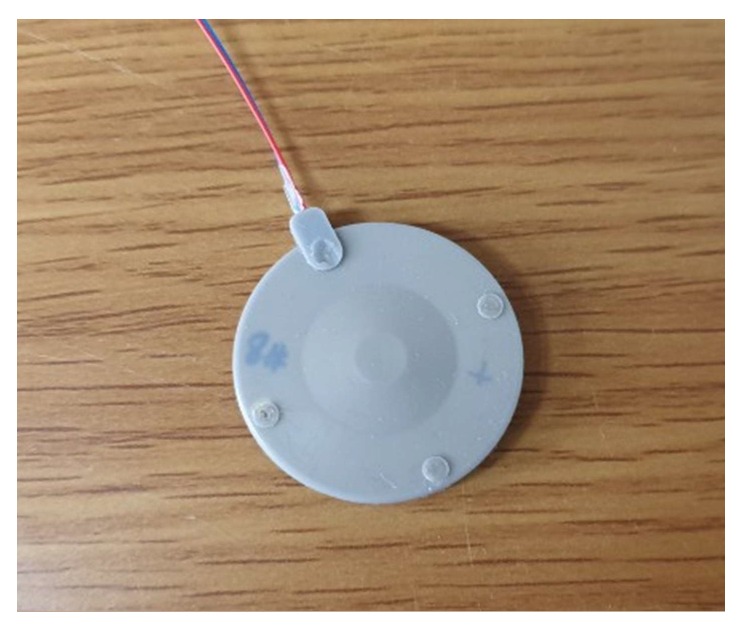
Photograph of the prototype cymbal transducer coated for measurement in water.

**Figure 13:**
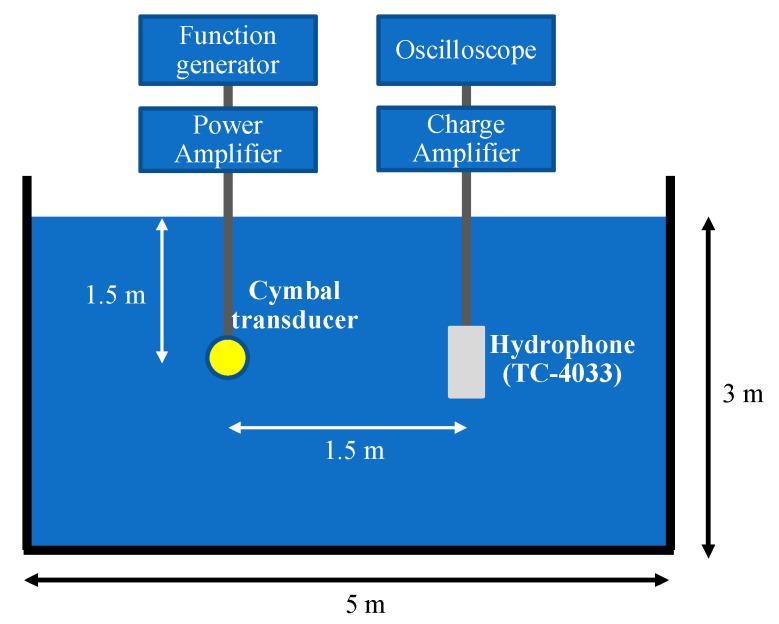
Schematic of the underwater TVR spectrum measurement setup.

After the measurement in air, the prototype transducer was coated with a thin layer of rubber (RTV-3460, Elkem, Norway) for evaluation of its underwater TVR characteristic. A fixture was used for the coating process and the thickness of the coating was controlled to be 0.25 mm. Figure 12 is a photograph of the coated prototype cymbal transducer. The underwater TVR spectrum of the transducer was evaluated with the measurement setup illustrated in Figure 13.

**Figure 14:**
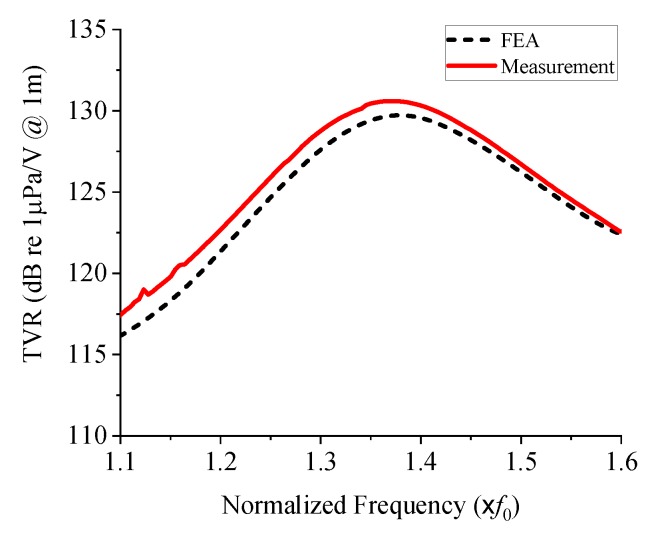
Comparison of simulated and measured TVR spectra of the prototype cymbal transducer.

The measured underwater TVR spectrum of the prototype cymbal transducer is presented in Figure 14, where it is compared with that analyzed with the FEA model. The two TVR spectra are almost identical; Table 4 presents a quantitative comparison of the two spectra. The difference in the peak TVR level is 0.7%, that of the center frequency is 0.1%, and that of the bandwidth is 2.8%. Even if small, the differences are thought to be mainly due to the effect of the rubber coating because the exquisite process of rubber mixing and curing is likely to have caused experimental errors. This comparison, in conclusion, verifies the validity of the optimal structure of the cymbal transducer and confirms that the wideband cymbal transducer can be designed with high accuracy by using the design method of this study.

## 5. Conclusions

In this study, the structure of a cymbal transducer was designed to have a wideband frequency characteristic by means of the FEM and the structural optimization technique. To achieve this, we analyzed the effect of various structural parameters on the transducer’s performance. Then, we optimized the structure of the transducer to have the widest possible bandwidth. We checked the validity of the design by fabricating a prototype of the optimized cymbal transducer and comparing its measured performance with simulation results. The impedance and underwater TVR spectra showed very good agreement between the measurement and simulation, which confirmed the accuracy and efficacy of the design method developed in this work. 

To the best knowledge of the authors, this is the first work to broaden the bandwidth of an individual cymbal transducer. The cymbal transducer designed in this work is different from existing transducers in that it can have both wide bandwidth and high TVR level performance. Broadband cymbal transducers can enable massive data transmission for underwater sensor network systems and improve the detection accuracy of sonar systems.

## Figures and Tables

**Table 1:** Structural parameters and initial dimensions of the cymbal transducer in Figure 3.

Structural Parameter	Symbol	Value (mm)
Thickness of the piezoceramic	*t_c_*	1.0
Thickness of the metal cap	*t_m_*	0.5
Height of the cavity	*h_c_*	0.5
Diameter of the cavity base	*d_b_*	15.4
Diameter of the cavity apex	*d_a_*	7.0
Diameter of the piezoceramic	*d_c_*	20.0
Diameter of the transducer	*d_ro_*	26.6
Width of the bonding layer	*w_b_*	0.3
Width of the metal ring	*w_r_*	3.0

**Table 2:** Optimized dimension of the cymbal transducer (unit: mm).

*t_m_*	*h_c_*	*d_b_*	*d_a_*
0.5	0.72	14.6	5.1

**Table 3:** Acoustic characteristics of the cymbal transducer before and after the structural optimization.

	Before Optimization	After Optimization
Peak TVR level (dB)	127.8	128.8
Center frequency (× *f*_0_)	1.0	1.38
Bandwidth (× *f*_0_)	0.14	0.24
Fractional bandwidth (%)	14.0	17.4

**Table 4:** Comparison of the measured and simulated TVR spectra of the prototype cymbal transducer.

	Measurement	FEA
Peak TVR level (dB)	130.6	129.7
Center frequency (×*f*_0_)	1.281	1.286
Bandwidth (×*f*_0_)	0.185	0.191
